# Supplementary material for: A network dynamical simulation model for the study of antibiotic resistance in microbial communities
Source: Gut Microbes. 2026 Sep 19;18(1):2734703. doi: 10.1080/19490976.2026.2734703 (PMC13596919; doi:10.1080/19490976.2026.2734703)
Supplement: Supplementary file 1 [file KGMI_A_2734703_SM9053.zip › supplementary_file_1/Figures/er_delta_density_n1000.pdf]

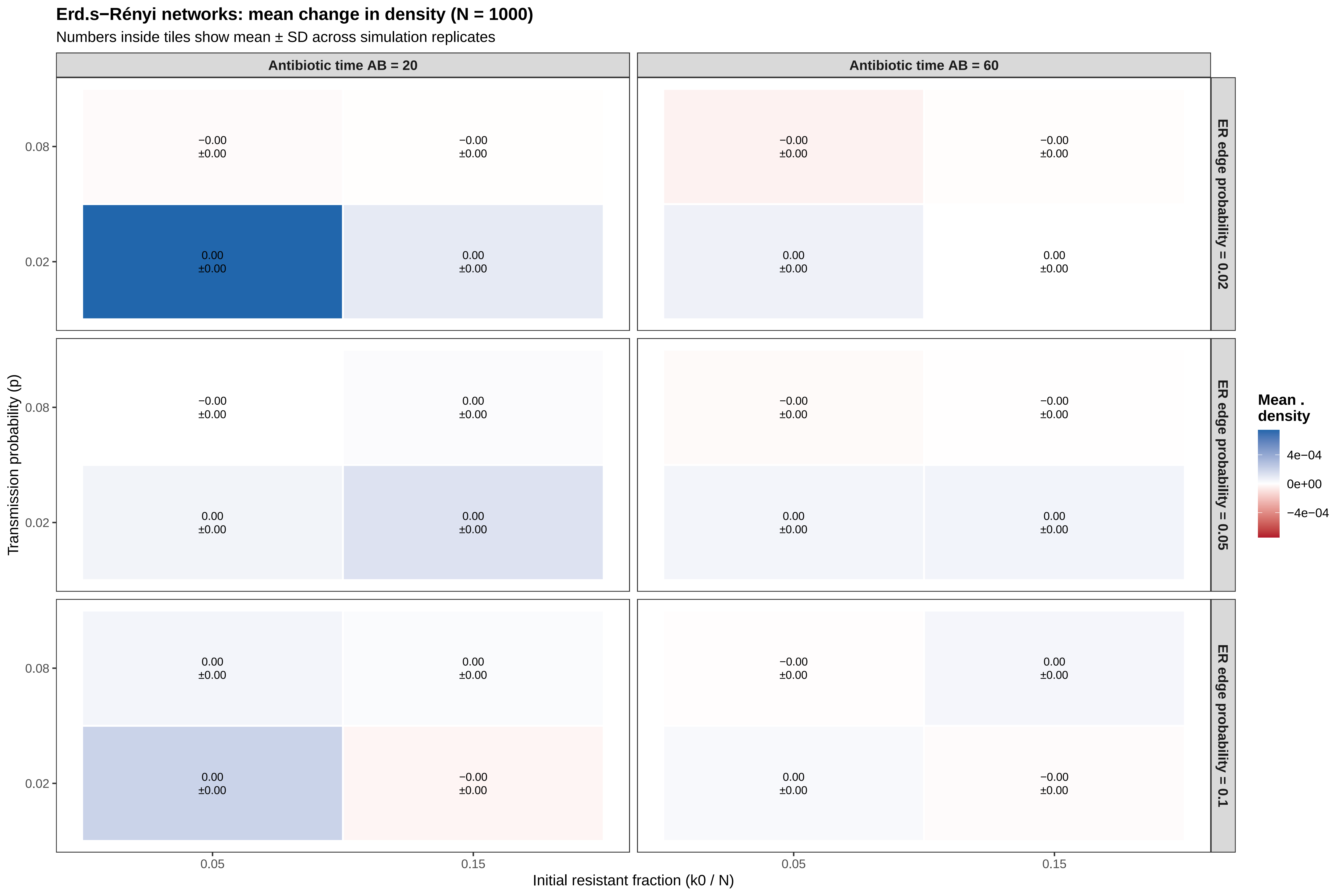
Erd.s−Rényi networks: mean change in density (N = 1000)
Numbers inside tiles show mean ± SD across simulation replicates
Antibiotic time AB = 20
Antibiotic time AB = 60
ER edge probability = 0.02
ER edge probability = 0.05
ER edge probability = 0.1
Transmission probability (p)
0.08
0.02
Initial resistant fraction (k0 / N)
0.05
0.15
−0.00
0.00
±0.00
Mean . density
4e−04
0e+00
−4e−04
